# Association between serum total cholesterol levels and Crohn’s clinical disease severity: a retrospective cross-sectional study

**DOI:** 10.3389/fmed.2025.1708838

**Published:** 2025-11-21

**Authors:** Jinchun Ni, Yunna Tang, Fangyuan Zhou, Yiqun Hu, Lupeng Liu, Mingcheng Huang, Hui Ouyang, Chenxi Xie

**Affiliations:** 1 Department of Gastroenterology, Clinical Research Center for Gut Microbiota and Digestive Diseases of Fujian Province, The National Key Clinical Specialty, School of Medicine, Zhongshan Hospital Xiamen University, Xiamen University, Xiamen, Fujian, China; 2 Xiamen Key Laboratory of Intestinal Microbiome and Human Health, Zhongshan Hospital Xiamen University, Xiamen, Fujian, China; 3 Department of Digestive Disease, School of Medicine, Institute for Microbial Ecology, Xiamen University, Xiamen, Fujian, China; 4 Department of Nephrology, Center of Kidney and Urology, The Seventh Affiliated Hospital, Sun Yat-sen University, Shenzhen, Guangdong, China; 5 Department of Digestive Medicine Center, The Seventh Affiliated Hospital, Sun Yat-sen University, Shenzhen, Guangdong, China

**Keywords:** Crohn’s disease, CDAI scores, serum total cholesterol, clinical disease activity, supplementary marker

## Abstract

**Background:**

This retrospective cross-sectional study aimed to investigate the relationship between serum total cholesterol (TC) levels and the clinical activity of Crohn’s disease (CD).

**Methods:**

One hundred and four patients with Crohn’s disease (CD) and twenty healthy volunteers were included in the analysis. Serum uric acid (SUA) levels and indicators related to lipid metabolism were measured within 1 week before undergoing endoscopic and CT enterography (CTE) examinations. Patients were divided into groups based on their Crohn’s Disease Activity Index (CDAI) scores.

**Results:**

Patients were categorized into mild and moderate groups, with no patients meeting the criteria for severe CD. The serum uric acid (SUA) and triglyceride (TG) levels were similar between CD patients and the control group (*p* > 0.05). However, the levels of total cholesterol (TC), apolipoprotein A1 (apo A1), apolipoprotein B (apo B), high-density lipoprotein cholesterol (HDL-C), and low-density lipoprotein cholesterol (LDL-C) were higher in the control group (*p* < 0.05). The TC and LDL-C levels were lower in the moderate patients compared to those in mild group (*p* < 0.05). TC ≤ 3.5 mmol/L was identified as an independent risk factor for more severe disease (OR = 4.50, 95%CI 1.612–12.561, *p* = 0.004). TC levels were correlated to both CRP and CDAI scores negatively (*p* < 0.05).

**Conclusion:**

TC may serve as a potential supplementary marker for clinical disease activity in CD, but further research, including longitudinal studies, is needed to confirm its reliability.

## 1 Introduction

Crohn’s disease (CD) is a chronic, recurring inflammatory bowel disease (IBD) that influences the whole digestive tract (1). Its severity can be evaluated by Crohn’s disease activity index (CDAI) values. But CDAI contains subjective evaluations and lacks stability (2). C-reactive protein (CRP) is commonly used to reflect mucosal inflammation, but its sensitivity still needs to be improved (3). Fecal calprotectin is better than CRP in clinical practice (3), but only qualitative testing is performed in some Chinese hospitals. It is still necessary to search for more cost-effective and readily available biomarkers related to CD inflammation.

The imbalance between prooxidants and antioxidants can lead to intestinal mucosal damage (4). Previous animal model study showed that oxidative stress could induce uric acid (UA) synthesis via activating Nrf2 pathway, and UA had a protective antioxidative effect on enterocytes in the intestinal lumen (5). But abnormally elevated UA may have opposite effects (6). The role of UA may be complex in inflammation. But only a few studies have focus on the association between CD and UA (7).

Abnormal lipid metabolism is a common feature in patients with inflammatory bowel disease (IBD). This may due to reduced daily food intake to avoid exacerbating gastrointestinal symptoms, malabsorption caused by chronic inflammation, enteric dysbacteriosis, and liver injury (8–12). Numerous studies have demonstrated a link between lipids and inflammatory disorders. However, findings regarding changes in lipid profiles among patients with IBD remain inconsistent. TC (total cholesterol) may decrease in IBD patients, but the changes of LDL-C (low-density lipoprotein cholesterol) and TG (triglyceride) are still controversial (13, 14). Due to the increased risk of cardiovascular disease in IBD patients, a decrease in HDL-C (high-density lipoprotein cholesterol) seems reasonable (13).

Among these parameters, TC may serve as the most reliable marker of inflammation in IBD. Previous studies showed that lower TC levels may accelerate IBD progression (15, 16). Reduced cholesterol levels may lead to excessive ATP consumption in enterocytes to sustain cholesterol synthesis, disrupt the metabolism of steroid hormones, and alter bile acid composition, thereby impacting gut microbiota balance. Given these multifactorial effects, further research into serum lipid changes may provide valuable insights into disease progression and management in IBD.

This research aims to enhance the understanding of lipid metabolism alterations in CD. Specifically, the study seeks to (1) compare the differences in SUA and serum lipid parameters between CD patients and controls, and (2) evaluate the potential associations between these indicators and the clinical activity of CD.

## 2 Materials and methods

### 2.1 Subjects

Consecutive patients admitted to our hospital for the first time and diagnosed with CD from January 2020 to January 2024 were enrolled in the retrospective study. Patients were excluded if they had the following conditions: unable to undergo colonoscopy and CT enterography (CTE) examinations due to severe intestinal obstruction or perforation; prior use of glucocorticoids, biologics, or lipid-lowering drugs; presence of gastrointestinal tumors; women who were pregnant or breastfeeding due to potential hormonal and metabolic changes; and severe renal, cardiac, or pulmonary disease, as these conditions would cause significantly dyslipidemia. Enrollment details are presented in the Results section. Twenty additional volunteers without lipid-lowering therapy, gastrointestinal symptoms, systemic disorders, or major abdominal surgery underwent colonoscopy to rule out subclinical intestinal disease and were included as healthy controls. Serum sampling, endoscopy and CTE examinations were completed in a week.

The study protocol and the recruitment of the patients were approved by the Ethics Committee of Zhongshan Hospital Xiamen University (Ethical approval No: xmzsyyky 2022-240). Written informed consent was obtained from all individuals before starting any study procedure. We confirmed that all methods were performed in accordance with the Declaration of Helsinki as revised in 2024.

### 2.2 Standards of grading for disease severity

The Crohn’s disease activity index (CDAI) was used to assess clinical disease severity (2). In this study, CDAI < 150 suggested remission, 150–220 with mild activity, 221–450 with moderate activity, and > 450 with severe activity.

### 2.3 Assessment of serum indicators

Samples were taken under fasting conditions. Seven indicators, including serum uric acid (SUA), LDL-C, HDL-C, TC, TG, apolipoprotein A1 (apoA1), and apolipoprotein B (apo B) were assessed in the study. Fecal calprotectin testing was not routinely available during the study period; therefore, CRP was selected as the inflammatory biomarker. All assays were performed according to the instructions of standard detection kits by an investigator blinded to the case status, ensuring that the evaluation of samples was not influenced by knowledge of whether they belonged to cases or controls.

### 2.4 Statistical analysis

Data are expressed as either the mean ± SD or the median (interquartile range). One-way ANOVA was used to compare differences if the values for a metric followed normal distribution; otherwise, the rank-sum test was used. ROC curve (receiver operator characteristic curve) was used to identify the thresholds of related indicators for distinguishing disease severity. Logistic regression analysis was applied to control confounding factors and investigate the association of lipid levels with clinical CD severity. The correlation between CDAI scores and TC was analyzed using the Spearman rank correlation coefficient. Similarly, the correlation between CRP and TC was assessed using the Spearman rank correlation coefficient. The *p-*value < 0.05 was considered statistically significant. The statistical analysis was accomplished using SPSS 24.0 (SPSS Inc., Chicago, IL, United States).

## 3 Results

### 3.1 Demographic characteristics of the patients

A total of 127 active CD patients who were first admitted to our hospital were screened. Nine patients unable to tolerate endoscopy or CTE examination and 11 patients with a history of intestinal surgery were excluded. One patient treated with prednisone and two patients treated with adalimumab were excluded. Ultimately, 104 patients were enrolled in the analysis (Table 1). Based on the CDAI scores, 24 patients were classified into the mild group. Eighty patients exhibited moderate activity, but none with severe disease (Table 2).

**TABLE 1:** Demographic data of the CD patients and controls.

Characteristics	Mild patients (*n* = 24)	Moderate patients (*n* = 80)	Controls (*n* = 20)	*P-*value
Age (years)	25.00**^a^** (20.25, 35.75)	28.00**^a^** (21.25, 33.00)	37 (32, 41)	< 0.01
Male (%)	79.17%**^a^**	75%**^a^**	40%	0.005
BMI (kg/m^2^)	20.26 (17.87, 23.91)	18.17**^a,b^** (16.37, 20.83)	22.94 (22.06, 25.36)	< 0.01

BMI, body mass index. For age and BMI: Kruskal-Wallis *H*-test. For gender distribution: Chi square test. The letter a means that the difference is significant when compared to the controls separately. The letter b means that the difference is significant when compared to the mild group separately.

**TABLE 2:** Comparison of serum indicators among patients classified by CDAI scores and controls.

Parameter	Mild patients (*n* = 24)	Moderate patients (*n* = 80)	Controls (*n* = 20)	*P-*value
SUA (μmol/L)	393.67 ± 101.01	358.07 ± 99.77	376.56 ± 109.77	0.299
TC (mmol/L)	3.83(3.50, 4.24)**^a^**	3.29(2.86,3.83)**^a,b^**	4.43(4.17,4.89)	< 0.001
TG (mmol/L)	1.17(0.85, 1.39)	0.92(0.68, 1.17)	1.15(0.77, 1.52)	0.101
Apo A1(g/L)	1.06(0.92, 1.21)^a^	0.98(0.83, 1.16)^a^	1.37(1.19, 1.55)	< 0.001
Apo B (g/L)	0.73(0.63, 0.86)	0.71(0.59, 0.84)^a^	0.88(0.79, 1.04)	0.013
HDL-C (mmo/L)	0.99(0.85,1.17)^a^	0.89(0.77, 1.08)^a^	1.35(1.17, 1.65)	< 0.001
LDL-C (mmo/L)	2.48(2.19, 2.83)	2.11(1.83, 2.52)^a,b^	2.94(2.53, 3.31)	< 0.001

SUA, serum uric acid; TC, total cholesterol; TG, triglyceride; Apo A1, apolipoprotein A1; Apo B, apolipoprotein B; HDL-C, high-density lipoprotein cholesterol; LDL-C, low-density lipoprotein cholesterol. For SUA: One-way ANOVA. For the other parameters: Kruskal-Wallis *H*-test *p* < 0.05 means that the distribution of values in each group is not equal. The letter a means that the difference is significant when compared to the controls separately. The letter b means that the difference is significant when compared to the mild group separately.

The age and gender distribution were similar between two CD groups (*p* > 0.05). The BMI was lower in moderate CD patients when compared to that of mild group (*p* < 0.01). This result was consistent with our previous conclusion (17).

### 3.2 Comparison of serum indicators between CD patients and control group

**FIGURE 1:**
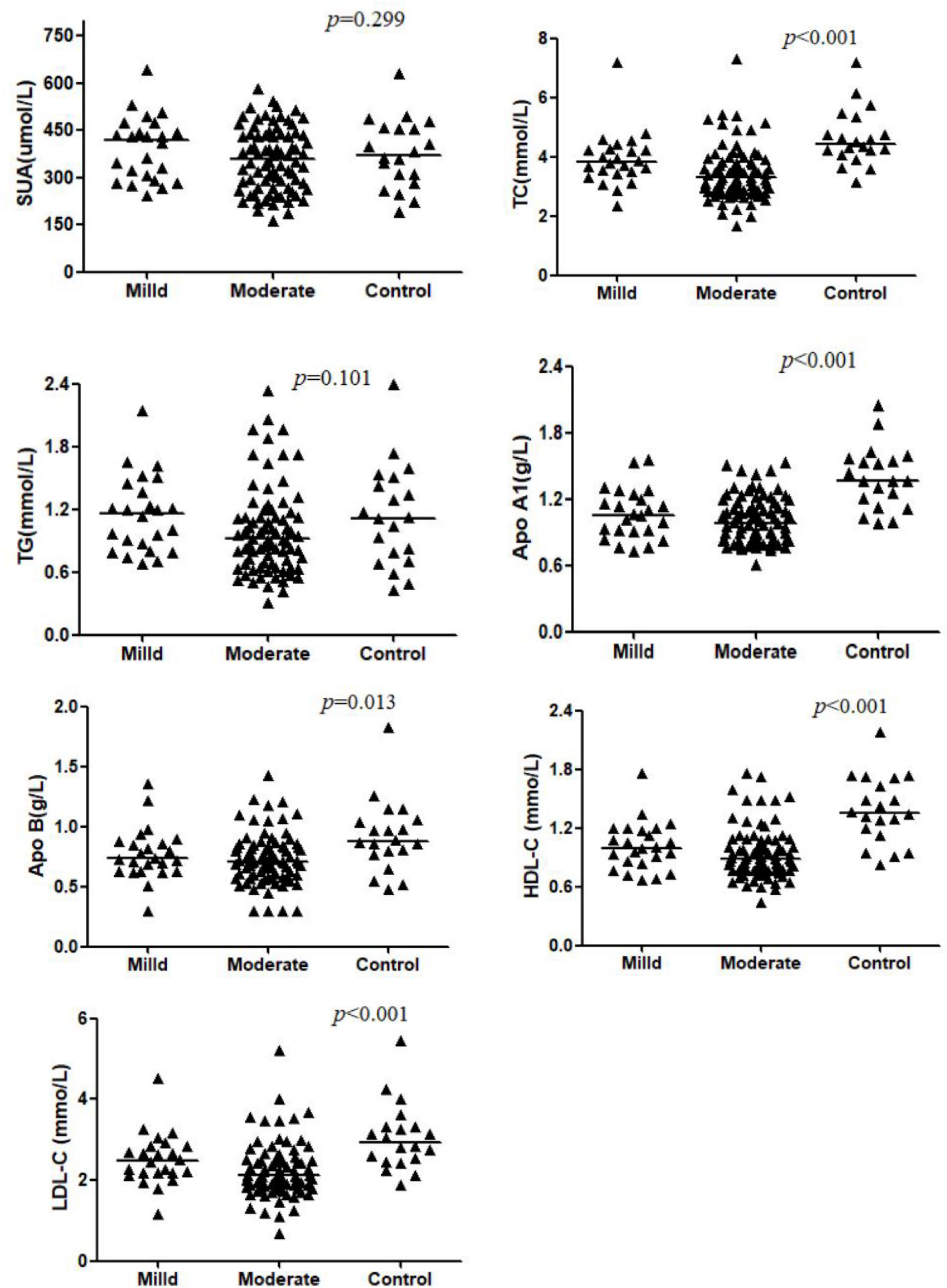
Comparison of serum levels of SUA, TC, TG, Apo AI, Apo B, HDL-C, and LDL-C across the Mild CD, Moderate CD, and Control groups. Data are presented as mean ± SD or median (interquartile range). *P*-values indicate the significance of the overall comparison among groups. The letter ‘a’ denotes a significant difference compared to the controls separately. The letter ‘b’ denotes a significant difference compared to the mild group separately.

The SUA and TG levels were comparable between CD patients and the control group (*p* > 0.05). Other parameters showed a decreasing trend in the CD groups (*p* < 0.05). However, the differences of apo B and LDL-C in the mild group were not significant when compared to that of controls (both *p* > 0.05). A significant difference in TC and LDL-C levels was observed between two CD groups (*p* < 0.05), while apo A1, apo B, HDL-C values were similar between mild and moderate patients (*p* > 0.05). These results were shown in Table 2 and Figure 1.

The statistic power for TC and LDL-C was 0.999 and 0.992, for HDL-C, apo A1 and apo B was 0.999, 0.999, and 0.868, for SUA and TG was 0.674 and 0.647.

### 3.3 Association of TC with clinical disease severity

**FIGURE 2:**
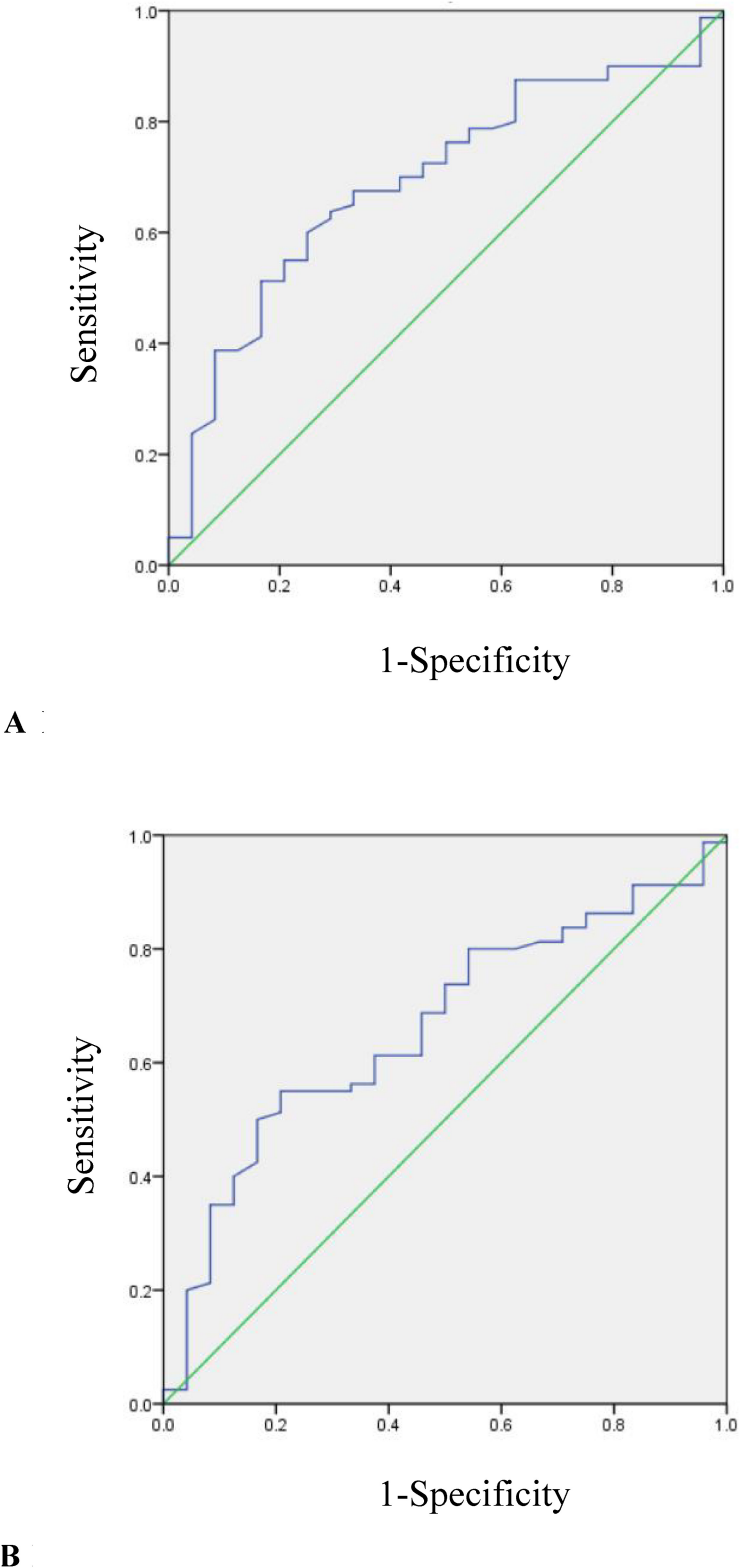
**(A)** ROC curve for determining the optimal threshold of TC in mild and moderate CD patients (AUC = 0.692, *p* = 0.004). **(B)** ROC curve for determining the optimal threshold of LDL-C in mild and moderate CD patients (AUC = 0.661, *p* = 0.017).

As only TC and LDL-C levels showed differences in two CD groups, they were further included in ROC analysis (Figure 2).

The cutoff value of TC used to distinguish moderate from mild group was 3.5 mmol/L (AUC = 0.692, *p* = 0.004). The sensitivity was 60%, the specificity was 75%.

The cutoff value of LDL-C used to distinguish moderate from mild group was 2.18 mmol/L (AUC = 0.661, *p* = 0.017). The sensitivity was 55%, the specificity was 79.2%.

The two cutoff values above were used to divide all the CD patients into two groups separately. A body mass index (BMI) < 18.5 kg/m^2^ was used as the demarcation of underweight (15). The comparison results of related parameters between mild and moderate CD patients were shown in Table 3.

**TABLE 3:** Comparison of metrics between CD patients with different disease severities.

Parameter	Mild patients (*n* = 24)	Moderate patients (*n* = 80)	*P*-value
Age (years)	25.00 (20.25, 35.75)	28.00 (21.25, 33.00)	0.521
Male (n)	19	60	0.675
BMI (<18.5 kg/m^2^)	8	42	0.099
TC (≤ 3.5 mmol/L)	6	48	0.003
TG (mmol/L)	1.15 ± 0.37	1.02 ± 0.47	0.226
SUA (μmol/L)	393.67 ± 101.01	358.07 ± 99.77	0.129
Apo B (g/L)	0.73(0.63, 0.86)	0.71(0.59, 0.84)	0.354
Apo A1 (g/L)	1.06(0.92, 1.21)	0.98(0.83, 1.16)	0.258
LDL-C (≤ 2.18 mmo/L)	5	44	0.003
HDL-C (mmo/L)	0.99(0.85, 1.17)	0.89(0.77, 1.08)	0.118

TC, total cholesterol; Apo A1, apolipoprotein A1; Apo B, apolipoprotein B; HDL-C, high-density lipoprotein cholesterol; LDL-C, low-density lipoprotein cholesterol.

No significant differences were observed between the mild and moderate CD groups regarding age, sex distribution, or the proportion of underweight individuals; however, the moderate group had a higher frequency of low TC and low LDL-C. We constructed a multivariable logistic regression model that included TC and LDL-C as primary predictors and adjusted for potential confounders (age, sex, and BMI). These results showed that lower TC was independently associated with more active disease (OR = 4.50, 95%CI 1.612–12.561, *p* = 0.004).

The TC were correlated to CDAI scores negatively (*r* = –0.263, *p* = 0.002), and was correlated to CRP negatively (*r* = –0.282, *p* = 0.004).

## 4 Discussion

Crohn’s disease is a chronic, intermittent inflammatory condition accompanied by various metabolic changes. Some of these metabolites may have anti-inflammatory effects, while others have pro-inflammatory effects (5, 6, 8, 18). Exploring the relationship between relevant serum indicators and IBD can help us better understand disease progression. In this study, we evaluated the association between blood lipid indicators, SUA and the severity of CD. We found that both TC and LDL-C was lower in patients with more severe disease. Lower TC was identified as an independent factor associated with moderate CD. This result was consistent with the findings of recent studies.

Intestinal tract is an important organ for UA production (19). H_2_O_2_ could stimulate the synthesis and secretion of UA in gut, with UA then alleviating the oxidative damage through Nrf2 pathway (5). Additionally, disruption of the commensal microbiota can lead to increased uric acid production and exacerbates colitis (20). Zhu F et al. reported a significantly increase of uric acid in IBD patients, but the serum uric acid to creatinine ratio (UA/Cr) was weekly correlated to disease activity (7). While many studies support that serum uric acid (SUA) increases during intestinal inflammation, Neubauer K et al found that systemic non-enzymatic antioxidant capacity was diminished and SUA decreased in IBD patients (4). SUA may not be a reliable biomarker for reflecting IBD activity. This aligns with our findings, as we did not observe significant changes in uric acid levels in patients with severe disease.

Endogenous cholesterol synthesis predominantly occurs in the liver, where acetyl CoA is converted into cholesterol molecules (21). Acetyl CoA, produced through the aerobic oxidation of carbohydrates, is crucial for the synthesis of butyric acid, one of three most common SCFA (short-chain fatty acids) in gut (22). Butyric acid levels are reduced in patients with inflammatory bowel disease (IBD), suggesting that acetyl CoA may be diminished in these patients (22). This was supported by our results. We found a decrease of TC, another key product of acetyl CoA, in CD patients. This metric was even lower in those with moderate disease.

Bile acids (BA) are synthesized from cholesterol in the liver, which is catalyzed by the cholesterol 7α-hydroxylase (CYP7A1) enzyme (23). Primary BAs are secreted to the gut after conjugation to glycine or taurine. They can be transformed into secondary BAs through the activity of gut microbiota (23). Activation of the farnesoid X receptor (FXR) exerts immunomodulatory and anti-inflammatory effects (24). This receptor can be directly activated in hepatocytes or by BAs in the small intestine. FXR negatively regulates hepatic BA synthesis by suppressing the expression of CYP7A1 (23, 25). In patients with inflammatory bowel disease, microbiota dysbiosis can lead to a decrease in the content of secondary bile acids (BAs). This induces a decrease of FXR and TGR5 (Takeda G-protein-coupled receptor 5), and enhances the transcription of NF-kB (23). Consequently, this exacerbates intestinal inflammation. Due to the reduced inhibitory effect of FXR, primary bile acids accumulate in the liver, leading to a decrease in cholesterol levels. Overexpression of CYP7A1 and low-cholesterol conditions can enhance the activation of SREBP2 (Sterol Regulatory Element-Binding Protein 2), a critical regulator of cholesterol metabolism (26). This activation upregulates the transcription of LDL receptor (LDL-R) (27). LDL-R on the hepatocyte surface facilitates the uptake of circulating LDL-C, thereby increasing intracellular cholesterol levels (23). Precious study highlights that transport of SCAP-SREBP2 complex from endoplasmic reticulum to Golgi apparatus is the key to optimal activation of NLRP3 inflammasome (28). Low blood cholesterol levels may impair the physiological synthesis of glucocorticoids, potentially exacerbating the progression of IBD (16). These findings underscore the intricate relationship between cholesterol metabolism and immune responses. In our study, serum TC and LDL-C levels were decreased in patients with moderate CD. However, only TC levels showed a correlation with disease activity. Although the associations between TC and CRP/CDAI were modest, they suggested that TC could potentially serve as a supplementary marker for monitoring CD inflammation.

In the study, Apo B, the main protein component of LDL, has been observed at lower levels in CD patients. Several factors may contribute to this phenomenon. First, during inflammation, interleukin-1 (IL-1) may suppress cholesterol synthesis, thereby reducing the secretion of both cholesterol and Apo B from liver (15, 29). Second, in severe disease, low-density LDL particles, which contain Apo B-100 and have lower TC content, are more prevalent (15, 30). These particles are also more prone to oxidation, which may further exacerbate lipid dysregulation.

Active Crohn’s disease can impair small intestine function, leading to reduced triglyceride production (15, 31). However, in our study, serum TG levels in CD patients were similar between mild and moderate CD groups, aligning with the findings of several other studies. Interestingly, TG levels may vary by sex in IBD patients, with an increase observed in males and a decrease in females compared to the general population (14). These variations suggest that TG may lack the consistency needed to serve as a reliable biomarker for disease activity.

During inflammation, a significant amount of serum amyloid A (SAA) was synthesized, which subsequently became the predominant apolipoprotein of HDL (32). SAA-enriched HDL can be taken up by macrophages rapidly or retained in adipose tissue, which may contribute to a reduction in serum HDL-C levels (15). The risk of cardiovascular disease may increase in patients with serious CD (33). HDL and its major apolipoprotein, apo A1, are known to provide cardiovascular protection, partly through the upregulation of endothelial nitric oxide synthase (eNOS) activity (34). Based on these mechanisms, it is expected that both HDL-C and apo A1 levels would decrease in patients with moderate Crohn’s disease. However, the differences between two CD groups were not significant in our study. This was consistent with the results of a recent Mendelian randomization study (16). Further research is needed to explore the mechanisms underlying changes in HDL-C levels in IBD.

There are some limitations in the study. First, the potential effects of age, BMI, and sex on lipid metabolism are acknowledged. Although the control group was not BMI-matched, our internal comparison focused on mild versus moderate CD. As shown in Table 3, there were no significant differences in age, sex distribution, or underweight proportion between these groups. Therefore, the association between lower TC levels and serious disease activity was unlikely to be substantially influenced by these factors. Second, the study was limited by a small sample size and the absence of patients with severe disease, which may restrict the generalizability of our findings. Future multicenter studies with larger cohorts should include patients across the full disease spectrum. Additionally, follow-up data after treatment were not available, and future longitudinal assessments of lipid levels across disease phases would help to further validate our results.

## 5 Conclusion

In summary, our findings indicate that decreased serum total cholesterol (TC) levels are associated with a more active inflammatory state in Crohn’s disease. TC may serve as a simple, inexpensive, and routinely available adjunct biomarker to complement existing indicators such as CRP and fecal calprotectin, especially in settings where advanced inflammatory assays are not readily accessible. Nevertheless, our results should be interpreted with caution and validated in large-scale, multicenter studies with long-term follow-up. Future research integrating fecal inflammatory markers, endoscopic assessments, and imaging correlations will be essential to further clarify the clinical significance of lipid metabolism parameters in Crohn’s disease.

## Data Availability

The original contributions presented in this study are included in the article/supplementary material, further inquiries can be directed to the corresponding author.
